# FDA-YOLO: A Feature Fusion and Attention-Based Network for Multiscale Tomato Maturity Detection in Real-World Agricultural Scenarios

**DOI:** 10.3390/s26113404

**Published:** 2026-05-27

**Authors:** Jiacheng Shi, Wenjun Luo, Xuemei Wang, Jian Guo, Hengyi Ren

**Affiliations:** 1 School of Computer Science, Nanjing University of Posts and Telecommunications, Nanjing 210023, China; b23041808@njupt.edu.cn (J.S.);; 2 Jiangsu Provincial Key Laboratory of Internet of Things Intelligent Perception and Computing, Nanjing University of Posts and Telecommunications, Nanjing 210023, China; 3 College of Information Science and Technology, Nanjing Forestry University, Nanjing 210037, China; renhy@njfu.edu.cn; 4 College of Artificial Intelligence, Nanjing Forestry University, Nanjing 210037, China

**Keywords:** deep learning, tomato, maturity recognition, FDA-YOLO, DCFA, multiscale detection, precision agriculture

## Abstract

Fruit detection and maturity recognition are crucial for intelligent tomato harvesting and management. However, in complex field environments, challenges such as the similarity in color between fruits and leaves, cluttered backgrounds, and severe occlusions significantly hinder accurate tomato detection. To address these issues, this paper proposes a lightweight tomato maturity detection model, termed FDA-YOLO. Building upon the YOLOv11 framework, the proposed model enhances global perception in complex scenarios by introducing a multiscale feature enhancement module. In addition, a foreground–background dual-path attention mechanism is designed to better distinguish fruits from the background, thereby improving detection robustness. Furthermore, a lightweight asymmetric detection head is constructed to reduce computational cost while maintaining high accuracy. These improvements enable the model to achieve more efficient and accurate tomato maturity detection under complex conditions. Extensive experiments are conducted on the LaboroTomato dataset. The results demonstrate that FDA-YOLO achieves the best performance with relatively low computational overhead, reaching 83.4% and 67.5% in mAP50 and mAP50–95, respectively, while also attaining a near-optimal F1 score. Overall, the proposed model achieves an excellent balance between accuracy and efficiency, providing an effective solution for intelligent agricultural monitoring and automated harvesting systems.

## 1. Introduction

Owing to their high nutritional value and distinctive flavor, tomatoes are among the most widely cultivated crops worldwide [1,2] and represent important sources of income and export revenue in many countries [3,4]. However, tomato harvesting still largely relies on manual labor and faces challenges, such as an aging agricultural workforce, labor shortages, and rising labor costs [5]. In addition, manual harvesting is labor-intensive, time-limited, and inefficient, making it difficult to meet the demands of large-scale modern agricultural production [6].

With the rapid development of smart agriculture [7,8], mechanical harvesting has become an important research focus [9]. Accurate fruit detection and maturity assessment are essential prerequisites for automated harvesting and directly affect harvesting efficiency, transportation and storage strategies, and final fruit quality and market value. Therefore, developing robust computer vision and deep learning methods for accurate real-time maturity detection is crucial for promoting intelligent tomato harvesting systems. Early approaches relied mainly on color thresholding [10], texture analysis [11], and morphological operations [12] but performed poorly under varying illumination and complex backgrounds. With the advancement of deep learning, convolutional neural networks (CNNs) have shown stronger adaptability in field environments because they learn hierarchical features in an end-to-end manner. As a result, deep learning-based detectors, such as Faster R-CNN [13], SSD [14], and YOLO [15], have become mainstream methods for tomato maturity recognition.

Despite recent progress, tomato maturity detection still faces several challenges. First, significant variations in fruit scale and distance, combined with complex illumination conditions, make it difficult to capture both the fine-grained textures of nearby fruits and the global shape information of distant fruits. Second, the similarity between fruit and leaf colors, together with occlusions and dense distributions, causes severe feature ambiguity, often leading to false detections. Finally, some detection heads suffer from structural redundancy and high computational cost, limiting real-time performance. Although some methods decouple classification and localization tasks, they still suffer from imbalanced feature utilization and excessive computational overhead, making deployment on resource-constrained devices difficult. Therefore, balancing detection accuracy, inference speed, and computational complexity remains a key challenge.

Existing YOLO-based improvements introduce mainly channel/spatial attention mechanisms and multiscale feature fusion strategies to enhance feature representation. However, most of these approaches rely on implicit feature reweighting and lack explicit foreground–background modeling while rarely considering joint optimization of feature modeling and detection structures. In complex agricultural environments, occlusion and background interference also remain insufficiently addressed, and some methods improve accuracy at the cost of higher computational overhead. Compared with recent attention-based YOLO variants, the fundamental novelty of our method is that the proposed DCFA module achieves explicit foreground–background dual-path decoupling and contrastive modeling, representing a paradigm shift from “implicit enhancement” to “explicit decoupling”.

To address these challenges, this study proposes FDA-YOLO, a lightweight and high-precision tomato maturity detection model based on YOLOv11 [16]. Unlike previous methods that focus solely on lightweight design or accuracy improvement, our approach aims to achieve high detection accuracy with low computational cost. The framework consists of three key components: focal spatial modulation (FSM), dual-contrast feature aggregation (DCFA), and an asymmetric depthwise separable detection head (ADSDH). The main contributions of this work are summarized as follows:

(1) **A lightweight and high-precision tomato maturity detection model, FDA-YOLO.** The proposed model is built upon YOLOv11 and is tailored to address challenges in tomato detection, such as scale variations and feature entanglement. It incorporates a more efficient feature modeling and detection mechanism, achieving a favorable balance between detection accuracy and computational efficiency. Consequently, the model is not only suitable for complex field environments but also deployable on resource-constrained embedded devices.

(2) **Introduction of focal spatial modulation (FSM).** To address the challenges related to scale variation, illumination differences, and the complex backgrounds of tomato fruits, we design an FSM module to enhance the model’s multiscale feature representation capability. FSM employs a hierarchical contextual focusing mechanism to modulate feature responses across different regions, effectively integrating local details with global semantic information. This approach enhances the overall feature representation of target fruits, enabling more accurate global perception in complex field environments.

(3) **Design of the dual-contrast feature aggregation (DCFA) module.** The DCFA module is proposed to address local feature confusion caused by color similarity between fruits and leaves, occlusions, and dense fruit distributions in agricultural scenarios. It leverages a foreground–background dual-path attention mechanism, combined with frequency-domain decomposition, to enhance salient features and complement information. By focusing on fine-grained local features and foreground–background separation, DCFA significantly improves the model’s discriminative ability and robustness for identifying tomatoes at different maturity stages. Compared with recent attention-based YOLO variants, the fundamental novelty of our method is that the proposed DCFA module achieves explicit foreground–background dual-path decoupling and contrastive modeling, representing a paradigm shift from “implicit enhancement” to “explicit decoupling”.

(4) **Development of the asymmetric depthwise separable detection head (ADSDH).** To overcome the issues of computational redundancy and uneven feature utilization observed in existing detection heads during multiscale feature fusion of high-resolution images, we design an ADSDH module based on the YOLOv11 detection head. This module decouples classification and localization tasks via an asymmetric branch design and replaces standard convolutions with depthwise separable convolutions. Such a design preserves detection accuracy while significantly reducing parameters and computational complexity, thereby enhancing real-time inference performance and efficiency on embedded platforms.

## 2. Related Work

### 2.1. Fruit Maturity Detection Based on Machine Learning Methods

Traditional detection approaches rely primarily on manually designed image features, such as color, size, shape, and texture, which are subsequently used by machine learning algorithms for fruit recognition and classification. The commonly employed algorithms for maturity detection include random forest [17], support vector machine (SVM) [18], and K-means clustering [19].

Zhou et al. [20] proposed a method for determining the maturity of field-grown red grapes based on an improved circular Hough transform, thereby providing a reference for harvest timing and automated picking. Wang [21] employed an HSI colour model that better aligns with human visual perception, combined with findContours and the equivalent diameter method, to classify tomato maturity and size. Huang et al. [22] utilized SVM to classify tomato maturity and achieved satisfactory recognition performance. Liu et al. [23] extracted histogram of oriented gradient (HOG) features to train an SVM classifier, thus enabling the preliminary detection of tomatoes at different maturity stages.

Although these traditional methods can achieve relatively good detection performance under controlled conditions, they are highly sensitive to factors such as illumination variation, fruit occlusion, and background complexity, making them less adaptable to the diverse conditions of natural field environments. Moreover, handcrafted features are insufficient to fully exploit the high-dimensional information in tomato images, including subtle local textures, nonlinear characteristics, and multiscale contextual relationships, which limits detection accuracy. Therefore, the practical applicability and generalization ability of these methods are constrained.

### 2.2. Deep Learning-Based Fruit Maturity Detection

With the development of deep learning, convolutional neural network (CNN)-based object detection methods have gradually replaced traditional algorithms that rely on handcrafted features and have become the mainstream approach for tomato maturity recognition. These models can automatically extract multilevel features through end-to-end learning, demonstrating stronger robustness and generalization ability. According to the detection pipeline, such methods are generally categorized into two-stage detection algorithms [24] and one-stage detection algorithms [25].

In two-stage detection algorithms, methods such as R-CNN [26], Fast R-CNN [27], and Faster R-CNN [13] first generate candidate regions and then perform classification and bounding box regression. Owing to their high detection accuracy and robustness, these algorithms have been widely applied in agricultural vision tasks. Rong et al. [28] optimized a Faster R-CNN VGG16-based model for tomato maturity grading by classifying tomatoes into unripe, half-ripe, and ripe categories, significantly improving the discrimination accuracy. Long et al. [29] modified Mask R-CNN to distinguish green, half-ripe, and fully ripe tomatoes and achieved favorable results. Yue et al. [30] employed an improved Cascade R-CNN network to classify green fruits and tomatoes at different maturity stages, enhancing the discrimination ability. However, this method relies on Cascade R-CNN with ResNet-101 as the backbone, resulting in high computational complexity and resource consumption during training and inference. In addition, it still has limitations in fine-grained discrimination between turning-stage and fully ripe tomatoes. Overall, although two-stage detectors provide high accuracy and robustness, their computational overhead is usually large. For example, CSPResNeXt-50 [31] and ResNet-101 [32] contain 20.50 M and 44.55 M parameters, respectively, which limits their application in real-time harvesting scenarios.

Compared with two-stage frameworks, one-stage object detection algorithms adopt a more streamlined architecture by integrating feature extraction, classification, and localization into a single network. These methods directly output object categories and bounding box locations in one forwards pass, eliminating the need for candidate region generation. Representative algorithms include the YOLO series and SSD, which achieve a better balance between detection accuracy and real-time performance. Owing to their fast processing capability for high-resolution agricultural images, one-stage algorithms have been extensively studied in terms of fruit recognition and maturity detection. Su et al. [33] proposed the SE-YOLOv3-MobileNetV1 network, which significantly improved the accuracy of tomato maturity classification. Lü et al. [34] modified YOLOv4 to differentiate tomatoes at various maturity stages. Chen et al. [35] conducted tomato maturity detection based on YOLOv5s, although missed detections still occurred in dense or heavily occluded scenarios. Han et al. [36] developed a lightweight YOLOv5-Lite model for papaya maturity detection, maintaining robustness under varying lighting and occlusion conditions. Zhang et al. [37] utilized YOLOv5-GAP to detect green grape clusters in dense and shaded environments. Peng et al. [38] proposed MFEFF-SSD for high-precision detection of small lychee targets in UAV images. Zhou et al. [39] combined YOLOv7 with traditional image processing methods to improve localization accuracy, although memory consumption increased. Wei et al. [40] developed GFS-YOLO11, which significantly improved multi-variety tomato recognition accuracy. Overall, compared with two-stage methods, one-stage detectors achieve a better balance between accuracy and speed. However, they remain sensitive to background interference and still have limited ability in terms of multiscale feature representation, which remains a major challenge.

In recent years, Transformer [41]-based self-attention mechanisms have demonstrated advantages in global feature interaction and object relationship modeling, making Transformer-based detection an emerging research direction in one-stage object detection. However, these methods still face challenges, such as slow training convergence, high memory consumption, and limited small-object detection capability, which restrict their practical deployment in agricultural scenarios.

Overall, deep learning-based fruit maturity detection methods demonstrate significant advantages in terms of automatic feature extraction and detection accuracy. Nevertheless, their complex structures, high computational overhead, and insufficient multiscale contextual modeling still limit their adaptability in complex field environments and lightweight deployment. To address these limitations, this study introduces multiscale feature modeling, feature modulation mechanisms, and a lightweight detection head within a one-stage framework to improve detection accuracy while maintaining computational efficiency.

### 2.3. Attention Mechanisms in YOLO-Based Detection

Attention mechanisms have been widely adopted to improve YOLO-based object detection models. For example, CBAM [42] recalibrates feature representations along both channel and spatial dimensions, and CA [43] incorporates coordinate information to enhance positional awareness. However, these methods are implicit feature enhancement approaches and do not explicitly distinguish between foreground and background information.

Some studies [21,44,45] have attempted to introduce foreground- and background-related attention mechanisms. For example, SEAM in YOLO-FaceV2 compensates for occluded regions by enhancing feature responses in unobstructed areas; the foreground dual attention in FCFPN captures foreground features from both channel and spatial dimensions. However, these methods essentially focus only on the foreground, lacking explicit modeling of background features, let alone explicit separation and contrastive interaction between foreground and background. Consequently, their effectiveness remains limited when addressing complex foreground–background confusion in tomato maturity detection.

To address this issue, our proposed DCFA module is designed with a foreground–background dual-path structure, enabling explicit feature separation and contrastive modeling and thereby more effectively mitigating this problem.

## 3. Methods

### 3.1. Overall Framework

**Figure 1:**
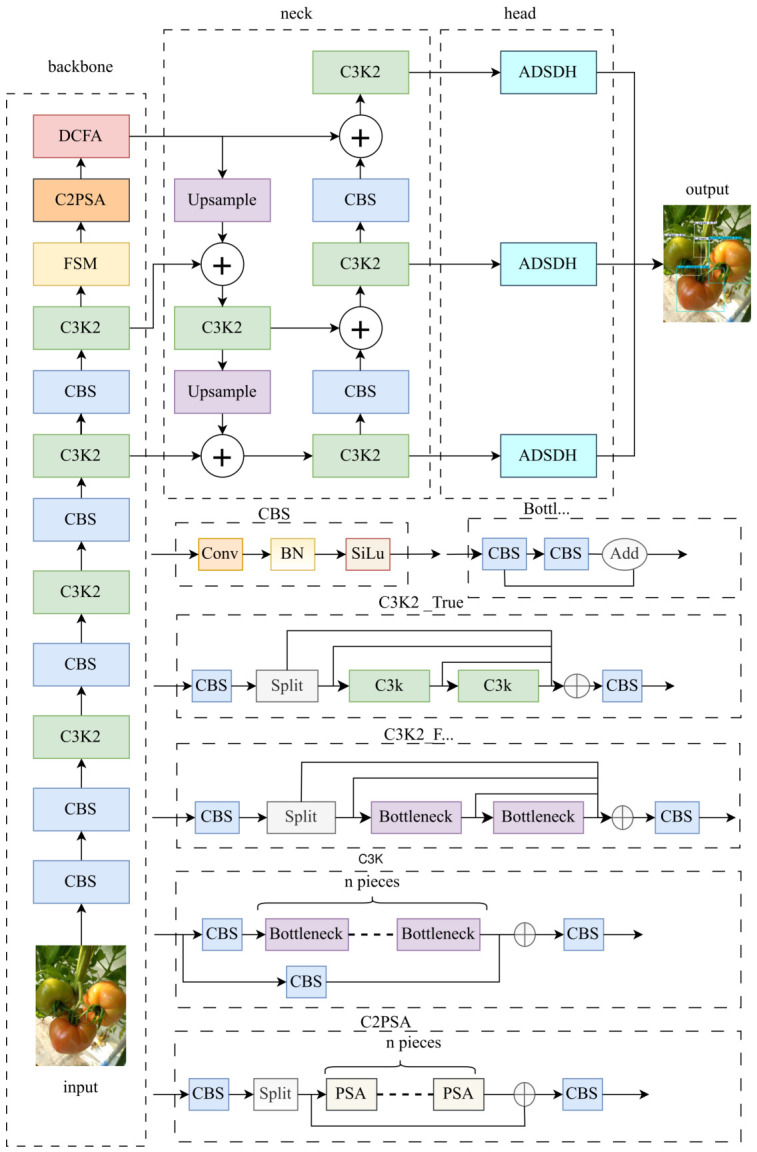
Overall framework of the proposed method.

This section provides a detailed description of the proposed tomato maturity detection model, **FDA-YOLO**, whose overall architecture is shown in Figure 1. The model is built upon the YOLOv11 framework released by Ultralytics on 30 September 2024 (YOLO Vision 2024) and specifically adopts the YOLOv11n (nano) version. It contains 238 layers, approximately 2.58M parameters, and 6.3 GFLOPs. All hyperparameters (e.g., learning rate of 0.01, momentum of 0.937, and weight decay of 0.0005) are kept consistent with the official Ultralytics benchmark settings to ensure fairness and reproducibility.

The choice of YOLOv11 as the baseline instead of the more established YOLOv8 is motivated by several considerations. Compared with YOLOv8, YOLOv11 introduces the C2PSA module, which incorporates spatial attention mechanisms to enhance the focus on critical regions, making it particularly effective for small and occluded object detection. In addition, the C3k2 module improves feature representation while reducing computational complexity. On the COCO dataset, YOLOv11m achieves a higher mAP than YOLOv8m does with approximately 22% fewer parameters. Furthermore, YOLOv11 has been applied in various domains, including underwater object detection [46,47], traffic detection [48], and metal defect detection [49], thus demonstrating its effectiveness and applicability.

FDA-YOLO consists of three main components: the backbone, neck, and head. Input tomato images are first fed into the backbone to extract multilevel semantic features, capturing both local texture details and global structural information. The neck then employs a multiscale feature pyramid structure (FPN + PAN) to fuse high-level and low-level features, enhancing the perception of fruits at different scales. Finally, the detection head outputs classification probabilities and bounding box locations for each fruit, enabling end-to-end maturity detection.

In the original YOLOv11 backbone, the spatial pyramid pooling fast (SPPF) module is used to enlarge the receptive field and enhance global feature modeling, achieving satisfactory performance in standard scenarios. However, in complex field environments, tomato fruits exhibit significant variations in scale, distance, and lighting conditions. Single-scale feature modeling struggles to capture both the fine details of nearby fruits and the global structure of distant fruits, limiting multiscale detection performance. To address this issue, the proposed method replaces SPPF with the FSM module to enhance multiscale feature representation.

Furthermore, owing to the high similarity in color and texture between tomato fruits and leaves, foreground and background features are easily confused in complex environments, and traditional attention mechanisms remain insufficient for distinguishing key regions. To address this issue, we place the DCFA module in the backbone, which leverages feature modulation and cross-scale information interaction to enhance foreground representation while suppressing redundant background interference, thereby improving robustness in occluded and dense scenarios.

With respect to the detection head, the original YOLOv11 structure still suffers from parameter redundancy and computational overhead under high-resolution inputs. Therefore, the proposed ADSDH module employs depthwise separable convolutions to reduce the number of parameters and computational cost. In addition, it decouples classification and regression tasks through an asymmetric structure, ensuring detection accuracy while maintaining lightweight efficiency.

In summary, FDA-YOLO inherits the efficiency of YOLOv11 while introducing targeted improvements in multiscale feature modeling, foreground–background discrimination, and lightweight detection head design, achieving a good balance between detection accuracy and computational cost. The following sections provide detailed descriptions of the FSM, DCFA, and ADSDH modules.

### 3.2. Focal Spatial Modulation

This section introduces the focal spatial modulation (FSM) module. This module is designed to enhance the representation capability of tomato fruits at different distances and scales while maintaining feature stability under complex illumination conditions. In tomato maturity detection, fruits exhibit significant scale variations: representations of nearby fruits contain rich texture details and subtle color gradients, whereas the detection of distant fruits relies mainly on shape and global color information. To address this issue, FSM replaces the SPPF module in YOLOv11. Although SPPF enlarges the receptive field through serial pooling, its feature fusion strategy is fixed, and it has a limited ability to model local details and channel dependencies. As a result, it struggles under complex lighting and background interference. The core idea of FSM is to achieve adaptive multiscale contextual fusion through a hierarchical convolution-gated aggregation mechanism, thus balancing global modeling ability and computational efficiency.

**Figure 2:**
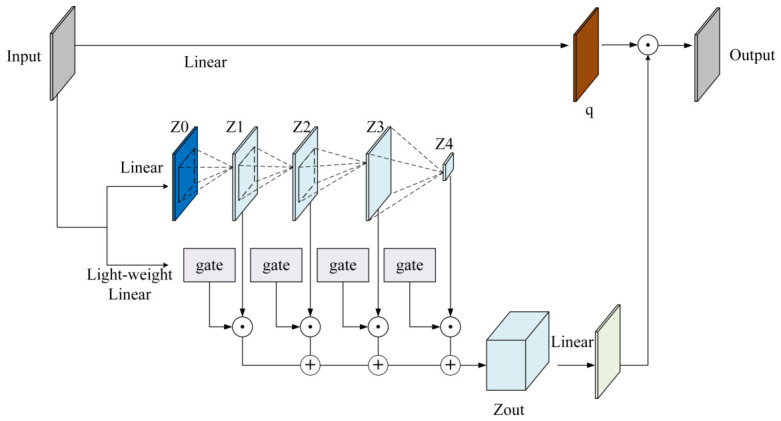
Architecture of FSM.

The workflow of FSM is shown in Figure 2. The module first applies linear mapping to generate a query feature. Then, multi-branch structures extract multiscale contextual information from different receptive fields. A gating mechanism is used to adaptively weight features from each scale. Finally, the fused contextual feature is combined with the original feature via elementwise multiplication, enhancing important regions while suppressing redundant information.

Specifically, let the input feature be $X\in R^{H\times W\times C}$, where *H*, *W*, and *C* denote the height, width, and channel number, respectively.

First, a linear projection is applied to generate the query feature *q*, initial contextual feature $Z_{0}$, and gating weights

(1) $$\left[q,\;Z_{0},\;\mathrm{gate}\right]=\mathrm{Split}\left(\mathrm{Linear}\left(X\right)\right),$$

where Split divides the feature along the channel dimension. $q\in R^{H\times W\times C}$ is the query feature, $Z_{0}\in R^{H\times W\times C}$ is the initial context feature, and $\mathrm{gate}\in R^{H\times W\times 4}$ controls the contribution of each scale.

Next, three stacked depthwise convolution (DWConv) layers are used to extract hierarchical contextual features $Z_{1}$, $Z_{2}$, and $Z_{3}$. Each layer applies a depthwise convolution with a kernel size $k_{l}$, followed by SiLU activation, and then SAAttention to refine important regions,

(2) $$Z_{l}=\mathrm{SA}\left(\mathrm{SiLU}({\mathrm{DWConv}}_{k_{l}}\left(Z_{l-1}\right))\right),\;l=1,\ldots ,3$$

where $Z_{l-1}$ denotes the output of the previous layer and $k_{l}$ represents the kernel size of the *l*-th convolution, covering receptive fields ranging from the local texture to the global structure. Small kernels focus on capturing fine-grained texture and local color variations of nearby fruits, whereas large kernels aggregate global semantics of distant fruits and background regions, thereby achieving a balance between “seeing clearly” and “seeing far”.

After hierarchical contextual extraction, the features from different scales are adaptively aggregated via a gating mechanism. Then, a weighted sum is calculated through elementwise multiplication to produce a feature map $Z_{\mathrm{out}}$ of the same size as the input *X*

(3) $$Z_{\mathrm{out}}=\sum_{l=1}^{L}{\mathrm{gate}}_{l}⊙Z_{l}+{\mathrm{gate}}_{L+1}⊙\left(\mathrm{AvgPool}(Z_{L})\right),$$

where ⊙ denotes elementwise multiplication. The second term corresponds to the global context path, aggregating the overall brightness and color distributions, which helps to mitigate feature shifts caused by varying illumination conditions. Although the gates corresponding to each scale are generated from the same linear mapping, they remain independent along the channel dimension and can adaptively modulate the response strength of different receptive field contexts based on the content of the input tomato image. This ability allows the effective selection and fusion of multiscale fruit information under complex field conditions. The gating aggregation mechanism enables the model to adaptively choose more reliable contextual features across scales, thereby enhancing the consistency of representations for fruits at different distances.

Finally, the aggregated contextual feature $Z_{\mathrm{out}}$ is passed through a linear mapping layer implemented with a 1×1 convolution and transformed into a modulation weight tensor. This tensor is then multiplied elementwise with the query feature *q* to perform adaptive feature modulation,

(4) $$Y=q⊙\mathrm{Linear}(Z_{\mathrm{out}}),$$

and the resulting output *Y* serves as the final enhanced feature of the FSM module and can be directly fed into subsequent network processing.

### 3.3. DCFA Module

This section introduces the proposed dual-contrast feature aggregation (DCFA) module, which is designed to enhance fine-grained fruit feature representation and improve foreground–background discrimination in complex field environments. In real-world scenarios, the strong similarity between fruit and leaf colors, occlusion, and dense fruit distribution often lead to feature confusion. To address this issue, DCFA performs frequency-domain decomposition and constructs a foreground–background dual-path attention mechanism to enhance the salient regions and compensate for missing information. Unlike FSM, which focuses on global feature modeling, DCFA emphasizes local fine-grained representations, thereby improving discrimination across different tomato maturity stages and enhancing robustness against background interference.

**Figure 3:**
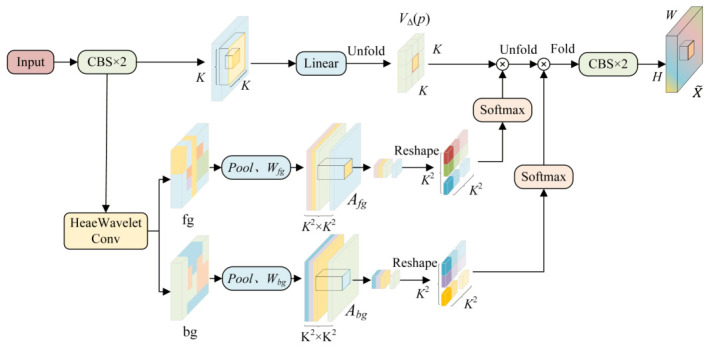
Architecture of DCFA.

The architecture of DCFA is shown in Figure 3. The processing pipeline consists of four stages: feature preprocessing with Haar wavelet decomposition, local unfolding, foreground–background dual-path attention modeling, and feature aggregation.

The first stage is feature preprocessing and Haar wavelet decomposition. Given the input feature map $X\in R^{H\times W\times C}$, two convolutional blocks are first applied to enhance the semantic representation,

(5) $$X^{\prime }=\mathrm{CBS}\left(\mathrm{CBS}\left(X\right)\right),$$

and the resulting feature $X^{\prime }$ is then decomposed using a Haar wavelet transform. Based on the discrete wavelet transform (DWT) [50], a single-level decomposition with four 2×2 Haar filters is applied, producing one low-frequency component, *a*, and three high-frequency components, *h*, *v*, and *d*. Here, *a* captures the global structure, smooth variation, and color distribution, while *h*, *v*, and *d* represent the horizontal, vertical, and diagonal texture details, respectively. In natural scenes, fruit regions contain richer high-frequency responses, while background regions are dominated by low-frequency information. Based on these properties, high-frequency components are grouped as foreground features (fg), and low-frequency components are treated as background features (bg), forming a dual-branch representation:

(6) $$\left(bg,fg\right)=\mathrm{HaarWaveletConv}\left(X^{\prime }\right)$$

The next stage is local unfolding. To model local relationships, the value feature *V* is first computed by applying a linear transformation with weight $W_{V}$ to the input feature $X^{\prime }$,

(7) $$V=W_{V}\left(X^{\prime }\right),$$

where $W_{V}\in R^{C\times C}$ and $V\in R^{H\times W\times C}$. Subsequently, for each spatial position, its local neighborhood is expanded. Specifically, an Unfold operation is applied to extract a K×K neighborhood centered at each position *p* and reshape it into a matrix $V_{\Delta }\left(p\right)$, where $V_{\Delta }\left(p\right)\in R^{K^{2}\times C}$. This matrix represents all pixel values within the local region centered at position *p*, which will be used for subsequent attention weighting.

Following local unfolding, foreground–background dual-path attention modeling is performed. The foreground and background feature maps are first processed by local average pooling and then passed through two independent linear layers, $W_{fg}$ and $W_{bg}$, to generate the foreground attention $A_{fg}$ and background attention $A_{bg}$, respectively,

(8) $$\left\{\begin{array}{c} A_{fg}=W_{fg}\left(\mathrm{Pool}\left(fg\right)\right)\\ A_{bg}=W_{bg}\left(\mathrm{Pool}\left(bg\right)\right) \end{array}\right.,$$

where Pool(·) denotes the local average pooling operation, $W_{fg},W_{bg}\in R^{C\times K^{4}}$, and $A_{fg},A_{bg}\in R^{H\times W\times K^{4}}$. The attention maps $A_{fg}$ and $A_{bg}$ are then reshaped along the last dimension, converting the local attention at each spatial position into vector form. A softmax function is subsequently applied to obtain normalized attention weights, denoted as $\mathrm{Softmax}(A_{fg})$ and $\mathrm{Softmax}(A_{bg})$.

Finally, feature aggregation is performed. For each spatial position *p*, the local features are subjected to a two-stage foreground–background weighting process. First, foreground attention is applied to emphasize the key local relationships in target regions, where the local features at each position are multiplied by foreground attention to obtain foreground-enhanced features. Then, these foreground-weighted features are expanded into neighborhood matrices via the Unfold operation and further multiplied by the background attention to incorporate structural information and enhance the understanding of complete semantics,

(9) $$V_{\Delta }^{\prime }\left(p\right)=\mathrm{Softmax}\left(A_{bg}\right)\left(p\right)⊗\left(\mathrm{Softmax}\left(A_{fg}\right)\left(p\right)⊗V_{\Delta }\left(p\right)\right),$$

where ⊗ denotes matrix multiplication. After aggregation over all positions, a Fold operation reconstructs the feature map back to $R^{H\times W\times C}$, followed by two CBS layers to produce the final output $\tilde{X}$.

Overall, DCFA enhances sensitivity to subtle differences in maturity while maintaining strong robustness under occlusion and dense distribution scenarios, thereby providing more stable and discriminative feature representations for the detection head.

### 3.4. ADSDH Module

This section introduces the asymmetric depthwise separable detection head (ADSDH) module. Existing detection heads typically adopt a unified branch design, which often overlooks the structural differences between classification and localization tasks when processing high-resolution fruit images and feature fusion, leading to insufficient feature utilization and increased computational burden. To address this issue, the ADSDH is designed based on the YOLOv11 detection head. By introducing differentiated structures for classification and regression branches and replacing standard convolutions with depthwise separable convolutions (DSConv), the proposed method constructs a lightweight and efficiently decoupled detection head. The ADSDH maintains detection accuracy while significantly reducing parameter count and computational complexity, thereby improving real-time inference capability on resource-constrained embedded platforms.

**Figure 4:**
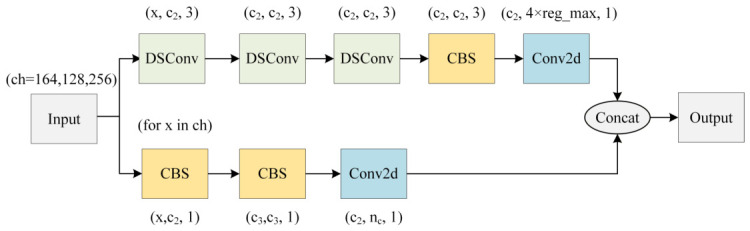
Architecture of the ADSDH.

The overall architecture of the ADSDH is shown in Figure 4. Its core idea is to further design task-specific branches with different depths for regression and classification on top of the original decoupled detection head, thus reducing redundant computations and improving task adaptability. Specifically, the regression branch focuses on accurate modeling of fruit boundary localization and spatial geometric structure, while the classification branch emphasizes the discrimination of color and texture features, thereby achieving a balance between detection performance and inference efficiency in resource-constrained scenarios.

In the ADSDH module, multiscale features from the backbone are first fed into the detection head. Let the input feature at the *i*-th scale be $X^{\left(i\right)}\in R^{B\times C_{i}\times H_{i}\times W_{i}}$, where i=1,2,3. These features are simultaneously passed to both regression and classification branches for localization and classification modeling, respectively.

The regression branch is relatively deeper and consists of stacked DSConv layers to enhance the modeling of scale variation and boundary information. The feature transformation is formulated as follows:

(10) $$R^{\left(i\right)}=\mathrm{Conv}\left({\mathrm{CBS}}_{1\times 1}\left(\mathrm{DSConv}(\mathrm{DSConv}\left(\mathrm{DSConv}\left(X^{\left(i\right)}\right)\right))\right)\right),$$

where $R^{\left(i\right)}\in R^{B\times C_{r}\times H_{i}\times W_{i}}$ denotes the regression feature at the *i*-th scale used for bounding box prediction. For each spatial location, the model predicts distance distributions to the four sides of the target bounding box (left, top, right, and bottom). Continuous boundary distances are then obtained via expectation over these discrete distributions, which are decoded into final bounding box coordinates.

To reduce the computational cost while preserving the representation ability, DSConv replaces standard convolutions. It decomposes convolution into depthwise convolution (DWConv) and pointwise convolution (1×1 Conv), where DWConv extracts spatial information and pointwise convolution performs channel fusion. This operation reduces complexity from $O(C_{in}C_{out}k^{2})$ to $O(C_{in}k^{2}+C_{in}C_{out})$, achieving lightweight computation without significant performance loss.

The classification branch focuses more on appearance-level color and texture cues, which are crucial for distinguishing maturity stages. Since classification requires weaker spatial modeling, this branch adopts a lightweight structure composed of stacked 1 × 1 Conv. The feature transformation is expressed as follows:

(11) $$C^{\left(i\right)}=\mathrm{Conv}\left({\mathrm{CBS}}_{1\times 1}\left({\mathrm{CBS}}_{1\times 1}\left(X^{\left(i\right)}\right)\right)\right),$$

where $C^{\left(i\right)}\in R^{B\times C_{c}\times H_{i}\times W_{i}}$ denotes the classification feature at the *i*-th scale.

Finally, the regression and classification outputs are concatenated along the channel dimension,

(12) $$Y^{\left(i\right)}=\mathrm{Concat}\left(R^{\left(i\right)},C^{\left(i\right)}\right),$$

where $Y^{\left(i\right)}\in R^{B\times \left(C_{r}+C_{c}\right)\times H_{i}\times W_{i}}$ is the fused detection feature at the *i*-th scale. The final bounding boxes and maturity categories are obtained through decoding.

The ADSDH achieves joint optimization of feature decoupling and a lightweight design. The regression branch focuses on spatial localization and boundary modeling, whereas the classification branch focuses on color and texture discrimination, effectively reducing intertask feature conflict. Moreover, by integrating DSConv, the ADSDH enables efficient real-time tomato maturity detection with significantly reduced computational cost while maintaining accuracy.

## 4. Experiments

We conduct experiments on the LaboroTomato dataset [51] to validate the effectiveness and superiority of the proposed FDA-YOLO algorithm.

### 4.1. Dataset

**Figure 5:**
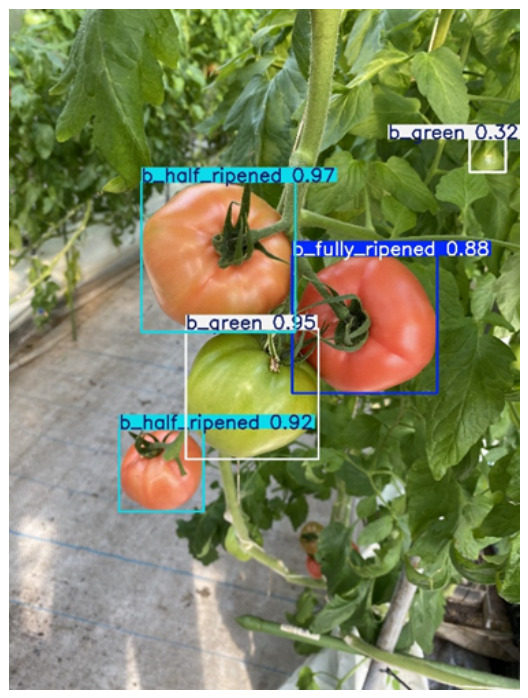
Examples of tomato detection results.

In this study, the LaboroTomato dataset is employed, which is a high-quality image dataset designed for tomato object detection and instance segmentation tasks, covering tomatoes at different maturity stages. The images are captured in orchard environments, with resolutions of 3024×4032 and 3120×4160 pixels. According to GH/T 1193-2021 [52], in combination with fruit growth conditions, tomato maturity is categorized into three stages: fully ripe, semiripe, and green, where the red coverage area exceeds 90%, with corresponding ranges of 30∼90% and 0∼30%, respectively. In addition, tomatoes are divided into large and small categories based on fruit size, resulting in a total of six classes. Examples of tomato detection results are shown in Figure 5. The dataset contains 804 images in total.

**Figure 6:**
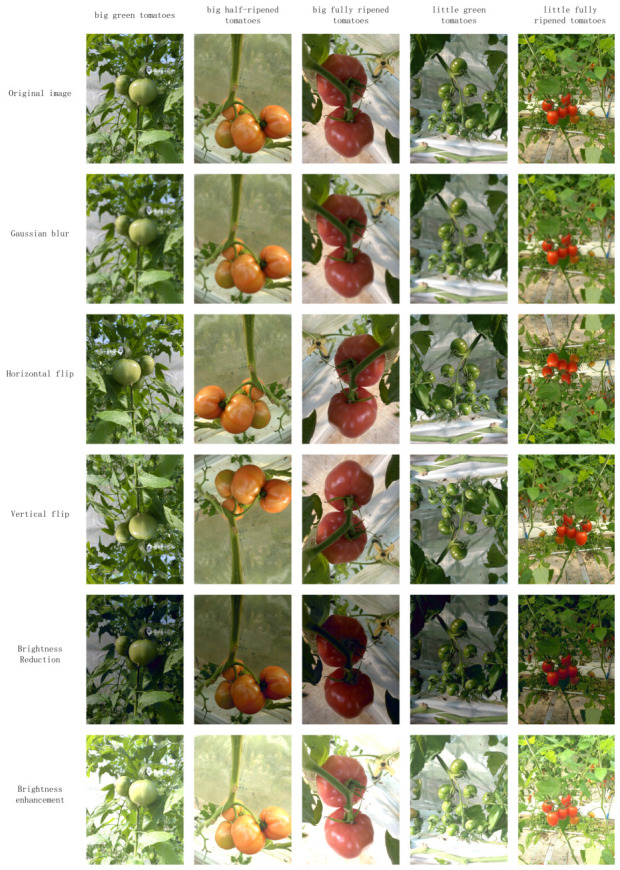
Examples of data augmentation techniques applied to the dataset.

Owing to the limited number of samples, data augmentation techniques are applied to increase data diversity and improve the generalization ability and robustness of the convolutional neural network in complex field environments. Specifically, Gaussian blur, horizontal flipping, vertical flipping, and brightness adjustment are employed, as illustrated in Figure 6. As a result, the dataset is expanded from 804 to 1608 images, which are then divided into training, validation, and test sets at a ratio of 7:2:1.

In addition, to standardize the input format and reduce the computational cost, all the images are resized to 640×640 pixels, which is required by YOLOv11. The reason is that larger image resolutions significantly increase training and inference time and may limit the network’s ability to effectively capture mid- and high-level features, thereby affecting detection performance [53]. In contrast, with this input size, the model benefits from a more consistent data distribution and more efficient feature processing, leading to improved computational efficiency and inference performance.

Finally, the preprocessed dataset is strongly representative in terms of fruit type, maturity level, and occlusion conditions, thus providing a reliable foundation for model training and evaluation.

### 4.2. Experimental Settings

The proposed model was implemented using PyTorch 2.5.1 and trained on an NVIDIA RTX 4090 GPU with 24 GB memory. Stochastic gradient descent (SGD) was adopted for optimization, and the input image size was set to 640×640. The hardware environment and training hyperparameter settings are summarized in Table 1.

### 4.3. Evaluation Metrics

To comprehensively evaluate the performance of the proposed model in terms of tomato maturity detection, the precision, recall, F1 score, mean average precision (mAP), parameters, GFLOPs, and FPS are adopted as evaluation metrics. These metrics assess detection performance from different perspectives. The precision reflects the prediction accuracy, the recall reflects the coverage of positive samples, and the F1 score balances the precision and recall. The mAP evaluates the overall detection performance across multiple categories. The parameters indicate the model size and storage requirements, GFLOPs reflects the computational cost per inference, and FPS reflects the real-time inference speed. The definitions of these metrics are presented below.

**Precision (P):** Precision represents the proportion of correctly predicted positive samples among all the predicted positive samples. Here, TP denotes true positive, and FP denotes false positive. It is calculated as follows:

(13) $$\mathrm{Precision}=\frac{TP}{TP+FP}$$

**Recall (R):** Recall represents the proportion of correctly identified positive samples among all actual positive samples, where FN denotes false negative. It is calculated as follows:

(14) $$\mathrm{Recall}=\frac{TP}{TP+FN}$$

**F1 Score:** The F1 score is the harmonic mean of the precision and recall, providing a balanced evaluation of prediction accuracy and completeness:

(15) $$F1=\frac{2TP}{2TP+FP+FN}$$

**Mean Average Precision (mAP):** mAP represents the mean value of the average precision (AP) across all classes in the dataset. Let AP(c) denote the average precision of class *c*, and let $N_{\mathrm{classes}}$ denote the total number of classes:

(16) $$\mathrm{mAP}=\frac{\sum AP(c)}{N_{\mathrm{classes}}}$$

In object detection tasks, mAP is typically evaluated under different intersection over union (IoU) thresholds. Specifically, mAP_50_ represents the average precision at IoU = 0.5, while mAP_50–95_ averages the results over IoU thresholds from 0.5 to 0.95 with a step size of 0.05, thus providing a stricter evaluation of localization performance.

**Parameters:** Parameters refer to the total number of trainable parameters in the model, reflecting its structural complexity and storage requirements.

**GFLOPs:** GFLOPs denote the number of floating-point operations required for a single forward inference. It directly reflects computational cost and inference efficiency.

**Frames Per Second (FPS):** FPS represents the number of image frames processed per second. It is an important metric for evaluating inference speed and real-time performance:

(17) $$\mathrm{FPS}=\frac{\mathrm{Total}\;\mathrm{Frames}\;\mathrm{Processed}}{\mathrm{Inference}\;\mathrm{Time}\;\left(\mathrm{seconds}\right)}$$

Unlike parameters and GFLOPs, FPS is affected by hardware configuration, input resolution, and optimization strategies, making it a more practical indicator of deployment performance.

### 4.4. Experimental Results and Analysis

To comprehensively validate the effectiveness and superiority of the proposed model and its key components, multiple groups of experiments are designed in this section, providing a progressive analysis from a macro perspective to a micro perspective. First, the overall performance of the model is evaluated through comparisons with several mainstream methods. Second, module replacement experiments are conducted to verify the effectiveness of the FSM module. Third, a dedicated comparison of different detection head structures is performed to demonstrate the advantages of the proposed design. Fourth, ablation studies are carried out to quantitatively analyze the individual contributions and synergistic effects of each component. In addition, we conducted a five-fold cross-validation experiment, a per-category performance analysis, and a generalization evaluation on different YOLO architectures to further verify the generalization ability and discriminative capability of the model.

#### 4.4.1. Comparison with Mainstream Models

To systematically evaluate the overall performance of the proposed model, experiments were conducted under a unified setting using YOLOv11n as the baseline. FDA-YOLO was compared with several mainstream models, including MobileNet [54], Swin Transformer [55], StarNet [56], InceptionNeXt [57], and different versions of the YOLO series. The results demonstrate the advantages of the proposed model in terms of both detection accuracy and computational efficiency.

The results are shown in Table 2. FDA-YOLO ranks first in terms of the two core metrics, mAP_50_ (83.4%) and mAP_50–95_ (67.5%), demonstrating strong and stable detection performance under different IoU thresholds. In terms of the F1 score, our method achieves 78.1%, which is only slightly lower than that of Swin Transformer-Tiny (78.8%). However, Swin Transformer-Tiny achieves this performance with 29.7 M parameters and 77.6 GFLOPs. In contrast, FDA-YOLO requires only 17.5% of the parameters and 9.8% of the computational cost while achieving superior mAP performance, with a marginal 0.7% F1 gap. This finding suggests that the slight advantage of Swin Transformer-Tiny results mainly from its larger model capacity rather than architectural efficiency. Compared with the other lightweight models, FDA-YOLO achieves the best performance in terms of recall, F1 score, mAP_50_, and mAP_50–95_ while ranking second in precision. These results demonstrate that the proposed model can extract features more effectively at a reasonable computational cost, thus significantly improving detection accuracy.

**Figure 7:**
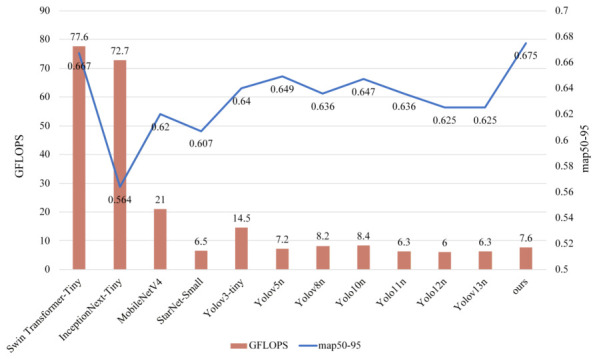
Comparison of different models in terms of accuracy and computational complexity.

To provide a more intuitive comparison, Figure 7 illustrates the distribution of different models in terms of detection accuracy and computational cost. FDA-YOLO achieves an excellent balance between accuracy and efficiency, outperforming mainstream detectors in terms of mAP_50_ and mAP_50–95_ while maintaining only 5.2 M parameters and 7.6 GFLOPs. Its computational cost is substantially lower than that of Swin Transformer-Tiny (29.7 M parameters and 77.6 GFLOPs). This favorable trade-off makes FDA-YOLO particularly suitable for resource-constrained scenarios, such as those involving UAVs and edge devices.

#### 4.4.2. Comparison of the FSM Module

To evaluate the effectiveness of the proposed focal spatial modulation (FSM) module, a comparative study was conducted within the YOLOv11 backbone. FSM was compared with three widely used feature enhancement modules: SPPF, SPP [58], and SPPF-LSKA [59]. For fairness, all the other network settings were kept consistent with the original YOLOv11 architecture and only the feature enhancement module was replaced.

As shown in Table 3, FSM maintains a lightweight design with only 2.7 M parameters and 6.4 GFLOPs while achieving the best performance on the key metrics. The precision, mAP_50_, and mAP_50–95_ reach 82.3%, 81.5%, and 66.2%, respectively, outperforming lightweight baselines such as SPPF. FSM improves mAP_50–95_ because its multiscale contextual modeling mechanism generates more accurate spatial responses for objects at different scales, thereby directly enhancing bounding box regression accuracy. The F1 score remains highly competitive. Although the recall (68.6%) is slightly lower, FSM still demonstrates superior overall detection performance while maintaining computational efficiency.

These results show that FSM effectively integrates multiscale contextual information and achieves strong detection performance with low model complexity, further demonstrating its practicality for real-world deployment.

#### 4.4.3. Comparison of the ADSDH Module

To validate the performance advantages of the ADSDH module in task decoupling and lightweight modeling, a comparative study was conducted under the same YOLOv11 backbone. The ADSDH was compared with SEAMHead [44], LQEHead [60], and detection heads with auxiliary branches [61]. For fairness, all the other network components were kept unchanged, and each comparison head retained its original design.

As shown in Table 4, the ADSDH achieves a precision of 79.9%, an F1 score of 76.3%, mAP_50–95_ of 81.6%, and mAP_50–95_ of 65.4%, outperforming all the compared detection heads. It also has the lowest model complexity, with only 2.3 M parameters and 5.2 GFLOPs. These results demonstrate that the ADSDH improves detection accuracy while maintaining high computational efficiency. Compared with LQEHead, the ADSDH further optimizes the classification and regression branches, achieving a better balance between feature extraction and localization accuracy. Its asymmetric depthwise separable design is particularly effective for multiscale features, improving the mAP while keeping the computational cost low.

#### 4.4.4. Ablation Study of the Proposed Modules

To quantitatively evaluate the contribution of each proposed module to the overall detection performance, a systematic ablation study was conducted based on the YOLOv11n baseline. As shown in Table 5, a controlled variable strategy was adopted through four progressive experiments. First, the FSM and DCFA modules were individually added to the baseline to evaluate their independent contributions. Then, both modules were introduced simultaneously to examine their combined effect. Finally, the ADSDH module was incorporated to form the complete model.

The results in Table 6 show that using only the FSM module increases mAP_50_ from 80.5% to 81.5% and mAP_50–95_ from 63.6% to 66.2%, demonstrating the effectiveness of multiscale contextual feature fusion. Introducing the DCFA module improves mAP_50_ to 81.9% and mAP_50–95_ to 65.7%, highlighting its ability to enhance local feature discrimination and foreground–background separation. When FSM and DCFA are combined, mAP_50_ further increases to 83.2%, while mAP_50–95_ reaches 67.6%, indicating a clear synergistic effect. After adding the ADSDH, the complete model achieves an F1 score of 78.1%, mAP_50_ of 83.4%, and mAP_50–95_ of 67.5%. Moreover, the computational cost is reduced from 8.8 GFLOPs to 7.6 GFLOPs, and the parameter count decreases from 5.5 M to 5.2 M. These results demonstrate that the ADSDH maintains detection accuracy while effectively reducing the overall computational burden, thereby achieving a better balance between accuracy and efficiency.

The performance improvement achieved by combining these modules can be attributed to their complementary functions. The FSM module enhances global contextual representation by modeling multiscale receptive fields, which improves feature extraction for objects of different scales. The DCFA module focuses on fine-grained feature enhancement and foreground–background separation through local contrastive modeling, thereby improving target discriminability. Finally, the ADSDH serves as a lightweight detection head that optimizes the decoupling of classification and regression branches while preserving the feature representation capability. As a result, it maintains detection accuracy while reducing the computational cost.

Therefore, FSM and DCFA improve feature quality from two different perspectives: global semantic modeling and local discriminative enhancement. Hence, they are functionally complementary rather than redundant. In contrast, the ADSDH performs structural optimization at the detection head stage and does not overlap with the feature enhancement modules in the backbone and neck. Instead, it further improves feature utilization efficiency.

The combination of these three modules enables the synergistic optimization of feature representation, object discrimination, and computational efficiency, ultimately improving overall performance. Through four progressive ablation experiments, this study validates the contribution of each module to detection performance and model efficiency. The results show that the proposed feature enhancement modules and improved detection head consistently improve the baseline performance while effectively controlling the parameter size and computational complexity, achieving a strong balance between accuracy and efficiency.

#### 4.4.5. Five-Fold Cross-Validation and Robustness Evaluation

To further evaluate the robustness and generalization ability of the proposed method, a 5-fold cross-validation experiment was conducted on the LaboroTomato dataset. Specifically, the original 804 images were divided into five mutually exclusive subsets. In each fold, four subsets were used for training, and the remaining subset was used for testing. This process was repeated five times, and the results were statistically analyzed.

To prevent data leakage, data augmentation was applied only to the training set. During each fold, Gaussian blur, horizontal flipping, vertical flipping, and brightness adjustment were adopted to increase the data diversity, while the test set remained unchanged.

The results of the 5-fold cross-validation are presented in Table 5. FDA-YOLO achieves stable performance across different data splits in terms of the precision, recall, F1 score, and mAP, with relatively small variations among folds. For example, mAP_50_ ranges from 81.3% to 84.9%, while mAP_50–95_ ranges from 66.5% to 70.4%, indicating that the model is not sensitive to specific data partitions.

Furthermore, compared with the baseline model, the proposed method consistently maintains performance improvements across all folds, demonstrating that it does not overfit a specific training subset.

Overall, the cross-validation results demonstrate the stability and certain robustness of the proposed method on the current dataset.

#### 4.4.6. Per-Category Performance Analysis Across Maturity Stages

To further evaluate the discriminative capability of the proposed method across different maturity stages, a categorywise performance analysis was conducted for all six classes, as shown in Table 7. Compared with the baseline YOLOv11n model, FDA-YOLO achieves performance improvements in most categories. In particular, categories such as b_fully_ripened and l_half_ripened show relatively significant improvements in both mAP_50_ and mAP_50–95_, indicating that the proposed model has a stronger recognition capability for categories with high visual similarity and greater classification difficulty. In addition, for categories that originally achieved relatively high performance, such as b_green and l_fully_ripened, the improved model maintains stable performance. This finding demonstrates that the proposed method enhances discriminative capability without sacrificing detection accuracy for easy-to-classify targets.

Overall, the results of the categorywise analysis demonstrate that the proposed method can effectively capture subtle differences among different maturity stages while alleviating multiscale detection challenges and foreground–background confusion to a certain extent. This finding further validates the effectiveness of FSM and DCFA.

#### 4.4.7. Qualitative Visualization Analysis

To further validate the effectiveness of the proposed method for multiscale detection and foreground–background discrimination, qualitative visualization comparisons between YOLOv11n and FDA-YOLO were conducted. Representative scenarios involving occlusion, complex lighting conditions, and scale variations were selected for evaluation.

**Figure 8:**
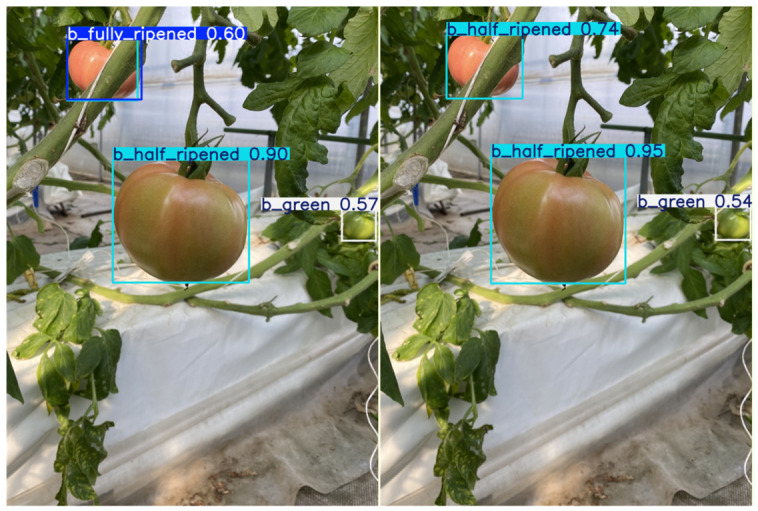
Detection comparison between YOLOv11n (**left**) and FDA-YOLO (**right**) (1).

As shown in Figure 8, the baseline YOLOv11n exhibits clear category confusion. Specifically, when a half-ripened tomato is occluded by vines, the baseline model misclassifies it as a fully ripened tomato with a relatively low confidence score (0.60). In contrast, FDA-YOLO produces more accurate and consistent predictions by correctly identifying the half-ripened tomato with higher confidence scores (0.95 and 0.74) while maintaining stable recognition of green tomatoes. These findings indicate that the proposed method is more effective in handling occlusion scenarios and further validate the role of the DCFA module in alleviating foreground–background confusion.

**Figure 9:**
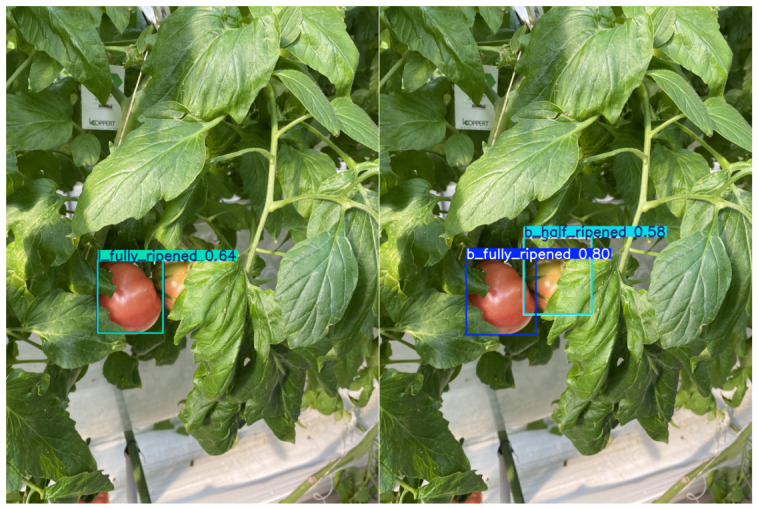
Detection comparison between YOLOv11n (**left**) and FDA-YOLO (**right**) (2).

As shown in Figure 9, this scenario is challenging because of complex lighting conditions and mutual occlusions among targets. The baseline YOLOv11n suffers from missed detections and category confusion, failing to detect one tomato target and producing an incorrect classification result (l_fully_ripened, 0.64). In contrast, FDA-YOLO successfully detects the missed target and corrects the misclassification. This difference demonstrates the effectiveness of the FSM module in handling scale variations among different tomato types (e.g., large and small fruits).

**Figure 10:**
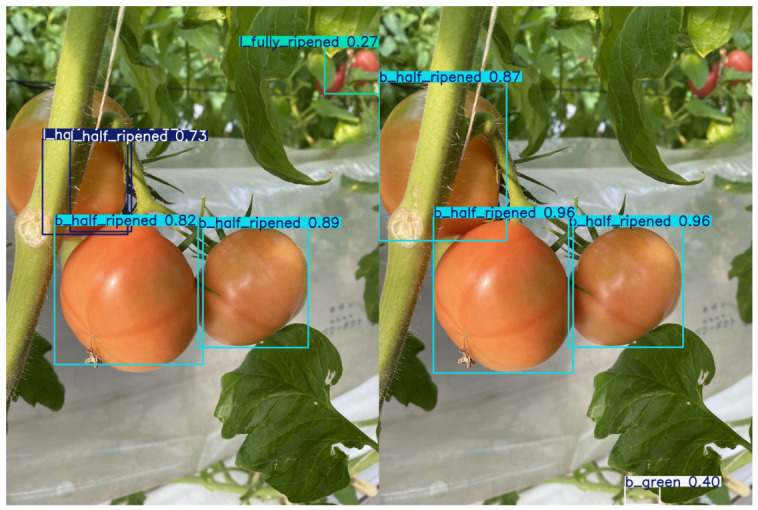
Detection comparison between YOLOv11n (**left**) and FDA-YOLO (**right**) (3).

As shown in Figure 10, a tomato is heavily occluded by vines. The baseline model fails to localize the target and cannot correctly identify its size category. In contrast, FDA-YOLO successfully localizes the target and accurately predicts both its size and maturity category. This difference further demonstrates the stronger robustness of the proposed method under complex occlusion conditions.

Based on the above qualitative results, the proposed framework offers several practical advantages for real-world agricultural deployment. First, FSM and DCFA collectively enhance detection robustness under challenging field conditions, such as leaf occlusion, illumination variation, and dense fruit clusters, directly improving the success rate of automated harvesting. Second, the lightweight ADSDH detection head enables real-time inference on resource-constrained edge devices, making the model suitable for agricultural robots and UAV platforms. Third, the method achieves significant improvements on visually similar and easily confused half-ripened tomato categories, providing fine-grained maturity assessment for optimal harvest timing and post-harvest grading.

#### 4.4.8. Generalization Evaluation

To evaluate the generality of the proposed method, we applied the proposed modules (FSM, DCFA, and the ADSDH) to another mainstream YOLO variant, YOLOv8n, under identical experimental settings. As shown in Table 8, our method brings consistent and stable improvements on both YOLOv8n and YOLOv11n. Specifically, for YOLOv8n, the proposed method achieves a precision of 81.7%, recall of 74.7%, F1 score of 78.0%, mAP_50_ of 82.9%, and mAP_50–95_ of 66.7%, which are significantly higher than the baseline. These results demonstrate that the proposed method is not specifically tailored to a single architecture but possesses good generalization capability across different YOLO versions.

## 5. Discussion

This study systematically investigates the design and optimization of a lightweight detection model. Overall, the results show that the proposed FDA-YOLO achieves a good trade-off between detection accuracy and computational efficiency. Specifically, FDA-YOLO outperforms the baseline model on key metrics, including precision, recall, mAP_50_, and mAP_50–95_, while maintaining stable performance across diverse unstructured natural scenes. These findings validate the effectiveness and superiority of the proposed method. All the reported gains are expressed as relative improvements.

FDA-YOLO consists of three core modules: FSM, DCFA, and the ADSDH. In the ablation study, the FSM module improves the ability of the baseline network to capture long-range dependencies through multiscale contextual extraction and gated aggregation. It yields a 1.2% improvement in mAP_50_ and a 4.1% improvement in mAP_50–95_, with only a slight increase in the computational cost. Notably, the DCFA module explicitly separates foreground and background features via Haar wavelet decomposition and adopts dual-path attention to model them independently, thereby enhancing target discriminability under occlusion and complex backgrounds. When combined with FSM, it increases mAP_50_ by 3.4% and mAP_50–95_ by 6.3% while keeping the computational cost manageable. The ADSDH further enhances feature discriminability while maintaining a lightweight structure. After FSM and DCFA are integrated, the F1 score improves by 2.5%, while the parameter count and FLOPs decrease by 5.5% and 13.6%, respectively, with mAP_50_ and mAP_50–95_ remaining largely stable.

FSM and DCFA improve accuracy but increase computational overhead, while the ADSDH reduces overhead through lightweight design. Overall, the three modules provide clear synergistic gains, enabling the proposed method to achieve a favorable trade-off between accuracy and computational cost, demonstrating strong efficiency and effectiveness. Considering the inference speed and resource constraints, the method is well suited for UAV inspection, field robotics, and edge computing devices, providing a practical solution for real-time deployment.

Despite these advantages, the proposed model has several limitations. First, for distant or very small fruits, limited feature information may reduce the detection accuracy. Second, the dataset covers a relatively limited range of scenes, which may affect generalization to more complex environments. Third, the model relies solely on RGB visual information without incorporating depth, spectral, or temporal cues, which may limit its applicability in broader agricultural scenarios. Finally, since all the experiments were conducted on a single dataset, a comprehensive assessment of the generalization capability of the proposed method still requires further cross-dataset validation in future work.

## 6. Conclusions

Accurate, real-time, and efficient object detection is crucial for autonomous tomato-picking robots during harvesting tasks. Although existing deep learning-based detection models achieve high accuracy, they are often limited by a substantial computational cost, large size, and high hardware demands, making deployment on resource-constrained agricultural robots or embedded platforms challenging. Moreover, environmental factors, such as varying illumination, occlusion, and differences in fruit maturity, further increase the complexity of the detection task. To address these challenges, this study proposes an improved FDA-YOLO model based on YOLOv11, incorporating FSM, DCFA, and ADSDH modules to facilitate feature extraction and detection head design. These enhancements strengthen global modeling capabilities, improve multiscale information fusion, and construct a lightweight detection head. The experimental results on an augmented dataset demonstrate that FDA-YOLO achieves precision, recall, F1 score, mAP_50_, and mAP_50–95_ of 81.3%, 75.2%, 78.1%, 83.4%, and 67.5%, respectively, while maintaining FLOPs and a parameter size of 7.6 and 5.2M, respectively. These results reflect a favorable balance between accuracy and efficiency, making the model particularly suitable for real-time deployment on embedded agricultural robots.

Future research directions include (1) optimizing the model architecture and hardware compatibility to further improve the inference speed and energy efficiency on resource-limited devices; (2) exploring multitask or multimodal information fusion, such as integrating depth, spectral, or infrared data to enhance robustness and applicability; and (3) extending the model to other crops or diverse environments to validate its cross-crop and cross-environment generalization abilities. These directions aim to broaden the practical application and deployment potential of the proposed model in intelligent agricultural systems.

## Figures and Tables

**Table 1:** Hardware environment and training parameter settings.

Items	Configuration
Operating System	Ubuntu 22.04.5 LTS (Jammy Jellyfish)
CPU	AMD EPYC 7542 32-Core Processor
Memory Size	32 GB
GPU (Memory Size)	NVIDIA GeForce RTX 4090 (24 GB)
CUDA Version	12.1
Python Version	3.10
Batch Size	32
Momentum	0.937
Epoch	300
Patience	20
Weight Decay	0.0005

All experiments were conducted under the above hardware and training configurations.

**Table 2:** Experimental results of different models on the dataset.

Models	Precision (%)	Recall (%)	F1 (%)	mAP_50_ (%)	mAP_50–95_ (%)	Params (M)	GFLOPs	FPS
MobileNetV4	71.5	74.5	73.0	78.0	62.0	5.4	21.0	87.2
Swin Transformer-Tiny	81.9	76.0	78.8	83.3	66.7	29.7	77.6	71.9
StarNet-Small	79.9	68.5	73.8	78.0	60.7	2.4	6.5	105.1
InceptionNeXt-Tiny	74.0	65.1	69.3	74.3	56.4	26.2	72.7	79.4
YOLOv3t	80.0	70.7	75.1	78.4	64.0	9.5	14.5	91.1
YOLOv5n	76.9	73.7	75.3	80.9	64.9	2.6	7.2	111.7
YOLOv8n	80.0	70.2	74.8	80.4	63.6	3.0	8.2	95.6
YOLOv10n	80.6	70.4	75.2	79.9	64.7	2.7	8.4	96.8
YOLOv12n	81.5	68.1	74.2	79.6	62.5	2.5	6.0	135.6
YOLOv13n	75.2	73.7	74.4	79.0	62.5	2.5	6.3	127.3
YOLOv11n	77.2	72.9	75.0	80.5	63.6	2.6	6.4	122.6
**Ours**	81.3	75.2	78.1	83.4	67.5	5.2	7.6	108.7

The best and second-best results are highlighted in red and blue, respectively, while the worst results are shaded in gray.

**Table 3:** Comparison of different focal modulation modules.

Models	Precision (%)	Recall (%)	F1 (%)	mAP_50_ (%)	mAP_50–95_ (%)	Params (M)	GFLOPs
SPPF	77.2	72.9	75.0	80.5	63.6	2.6	6.4
SPP	71.3	71.5	71.4	76.9	58.8	2.6	6.4
SPPF-LSKA	77.2	72.8	74.9	80.4	64.2	2.9	6.7
**FSM**	82.3	68.6	74.8	81.5	66.2	2.7	6.4

The best results are highlighted in red.

**Table 4:** Comparison of different detection heads.

Models	Precision (%)	Recall (%)	F1 (%)	mAP_50_ (%)	mAP_50–95_ (%)	Params (M)	GFLOPs
SEAMHead	74.2	73.7	73.9	79.3	61.3	2.5	5.9
LQEHead	78.1	72.3	75.1	80.7	64.6	2.6	6.5
aux	76.7	73.4	75.0	79.9	64.3	3.0	8.2
**ADSDH**	79.9	73.0	76.3	81.6	65.4	2.3	5.2

The best results are highlighted in red.

**Table 5:** Five-fold cross-validation results of FDA-YOLO.

Fold	Precision (%)	Recall (%)	F1 (%)	mAP_50_ (%)	mAP_50–95_ (%)
fold1	78.8	74.7	76.7	81.3	66.5
fold2	80.0	78.3	79.1	84.9	70.4
fold3	80.1	72.6	76.2	82.3	67.0
fold4	76.3	76.0	76.1	82.5	66.6
fold5	81.6	76.6	79.0	84.6	68.7
**Avg**	**79.4**	**75.6**	**77.4**	**83.1**	**67.8**

Average performance across all folds is reported in bold.

**Table 6:** Ablation study of the proposed components.

Methods	FSM	DCFA	ADSDH	P	R	F1	mAP_50_	mAP_50–95_	Params (M)	GFLOPs
YOLOv11n	–	–	–	77.2	72.9	75.0	80.5	63.6	2.6	6.4
YOLOv11n	✓	–	–	82.3	68.6	74.8	81.5	66.2	2.7	6.4
YOLOv11n	–	✓	–	79.6	73.2	76.3	81.9	65.7	5.4	8.6
YOLOv11n	✓	✓	–	78.1	74.4	76.2	83.2	67.6	5.5	8.8
YOLOv11n	✓	✓	✓	81.3	75.2	78.1	83.4	67.5	5.2	7.6

P: precision; R: recall. The best and second-best results are highlighted in red and blue, respectively, while the worst results are shaded in gray. ‘–’ indicates the module is not used; ‘✓’ indicates the module is used.

**Table 7:** Comparison of mAP performance across six categories (YOLOv11n → FDA-YOLO).

Categories	mAP_50_ (%)	mAP_50–95_ (%)
b_fully_ripened	77.0 → 80.0	61.5 → 68.3
b_half_ripened	78.3 → 84.8	59.0 → 69.8
b_green	90.0 → 90.0	70.5 → 70.8
l_fully_ripened	80.6 → 82.0	67.3 → 67.4
l_half_ripened	76.8 → 80.0	62.1 → 65.2
l_green	80.5 → 83.8	61.1 → 63.5

Significant improvements are highlighted in red.

**Table 8:** Generalization evaluation on different YOLO architectures.

Model	Precision (%)	Recall (%)	F1 (%)	mAP_50_	mAP_50–95_ (%)
YOLOv8n	80.0	70.2	74.8	80.4	63.6
YOLOv8n + Ours	81.7	74.7	78.0	82.9	66.7
YOLOv11n	77.2	72.9	75.0	80.5	63.6
YOLOv11n + Ours	81.3	75.2	78.1	83.4	67.5

Results improved by our method are highlighted in red.

## Data Availability

The dataset used in this study is the publicly available Laboro Tomato dataset, which can be accessed via GitHub (https://github.com/laboroai/LaboroTomato, accessed on 1 March 2026) and Kaggle.
